# Abdominal obesity and prostate cancer risk: epidemiological evidence from the EPICAP study

**DOI:** 10.18632/oncotarget.26128

**Published:** 2018-10-02

**Authors:** Céline Lavalette, Brigitte Trétarre, Xavier Rebillard, Pierre-Jean Lamy, Sylvie Cénée, Florence Menegaux

**Affiliations:** ^1^ Université Paris-Saclay, Université Paris-Sud, CESP (Center for Research in Epidemiology and Population Health), Inserm, Team Cancer and Environment, Villejuif, France; ^2^ Registre des Tumeurs de l’Hérault, ICM, Montpellier, France; ^3^ Service Urologie, Clinique Beau Soleil, Montpellier, France; ^4^ Institut Médical d’Analyse Génomique-Imagenome, Montpellier, France

**Keywords:** prostate cancer, obesity, waist circumference, waist-hip ratio, body mass index

## Abstract

Obesity is associated with an increased risk of several cancers, but inconsistent results have been observed between body mass index (BMI) and prostate cancer (PCa) risk. However, some associations have been reported with other indicators such as waist circumference (WC) and waist-hip ratio (WHR). We investigated the role of anthropometric indicators in PCa risk based on data from the Epidemiological study of Prostate Cancer (EPICAP).

EPICAP is a population-based case-control study that included 819 incident PCa in 2012–2013 and 879 controls frequency matched by age. Anthropometric indicators (weight, height, WC, and hip circumference) have been measured at interview. Logistic regression models were used to assess odds ratios (ORs) for the associations between anthropometric indicators (BMI, WC and WHR) and PCa risk.

We observed a slight, but not significant increased risk of PCa for men with a WC > 94 cm (OR 1.20, 95% CI 0.92–1.56) and for men with a WHR ≥ 0.95 (OR 1.30, 95% CI 1.00–1.70 between 0.95 and 1.00, OR 1.25, 95% CI 0.96–1.61 above 1.00). Associations were more pronounced after adjustment and stratification for BMI and in men with aggressive PCa.

Our results suggest that abdominal obesity may be associated with an increased risk of PCa, especially aggressive PCa.

## INTRODUCTION

Prostate cancer (PCa) is the most common male cancer in western countries with more than one million men diagnosed with prostate cancer in 2012 worldwide [1]. In France, more than 50,000 prostate cancer cases were diagnosed in 2011, with almost 9,000 deaths which represents the third cause of cancer-related mortality [2, 3]. Except age, ethnic origin, and family history of prostate cancer that are well-established non modifiable risk factors, the etiology of prostate cancer remains largely unknown.

Obesity has been associated with an increased risk of several cancers, including breast in post-menopausal women, endometrium, kidney, colon, and pancreas [4, 5]. However, the link between obesity and prostate cancer is still under debate, with inconsistent results across studies and according to the indicators used to characterize obesity. An extensive literature, almost 80 studies, has focused on body mass index (BMI), and null or weak results have been reported, as showed in several meta-analyses [5–9]. Nevertheless, out of the five meta-analyses published on BMI and prostate cancer, only two, which represents less than 25 studies overall [7, 8], were able to distinguish the aggressiveness of prostate cancer showing positive associations between BMI and aggressive prostate cancer. The lack of epidemiological evidence between BMI and prostate cancer is questionable, while some positive associations have been reported with other anthropometric indicators, such as waist circumference (WC) or waist-hip ratio (WHR) [10–19]. Therefore, it has been hypothesized that BMI itself is not the adequate indicator to capture obesity as it is influenced by both adipose and non-adipose tissue and does not take into account adipose distribution (i.e. abdominal or peripheral adiposity). It is more likely that abdominal obesity indicators (i.e. WC and WHR) independently, or combined with BMI, would better capture the concept of body fat distribution [10].

Several biological mechanisms have been proposed to understand how obesity may be related to prostate cancer. First, obesity may be correlated with a low physical activity level, suspected to increase the risk of prostate cancer [20]. Second, obese men have higher levels of insulin and insulin-like growth factor [21, 22], thought to promote carcinogenesis and inhibit apoptosis [23–25]. Finally, experimental and epidemiological studies also suggested that chronic inflammation may be associated either with initiation or progression of several cancers, including prostate cancer [26–31]. Indeed it has been suggested that obesity confers a low-grade inflammation status that may contribute to cancer development.

In that context, we aimed to identify modifiable risk factors exploring associations between several anthropometric indicators and prostate cancer, using data from the Epidemiological study of Prostate Cancer (EPICAP).

## RESULTS

The characteristics of prostate cancer cases and controls are presented in Table 1. Among prostate cancer cases, 77.3% were categorized as low or intermediate aggressive cancer and 22.7% as aggressive cancer. Age in 5-year groups was similarly distributed between cases and controls (*p* = 0.14). The EPICAP study population was mainly Caucasian (≥ 97%, *p* = 0.41), and as expected, family history of prostate cancer in first-degree relatives was more frequent in cases than in controls (24.8% and 9.6%, respectively) (*p* < 0.0001). Considering sociodemographic and lifestyle characteristics, cases and controls were similar in terms of educational level, smoking status and alcohol consumption (*p* = 0.27, *p* = 0.21, *p* = 0.22 respectively). Personal history of cardiovascular diseases (myocardial infarction, angina pectoris, stroke) was similarly distributed between cases and controls (*p* = 0.64).

**Table 1:** EPICAP study population characteristics

	Cases *n* = 819 (%)	Controls *n* = 879 (%)	*p*-value^1^
Gleason score			
<7	341 (42.3)	-	
7 (only 3+4)	282 (35.0)	-	
≥7 (including 4+3)	183 (22.7)	-	
Age (years)			0.14
<55	48 (5.8)	59 (6.7)	
55–59	99 (12.1)	99 (11.3)	
60–64	217 (26.5)	201 (22.9)	
65–69	274 (33.5)	285 (32.4)	
≥70	181 (22.1)	235 (26.7)	
Ethnic origin			0.41
Caucasian	795 (97.0)	859 (98.0)	
Others	24 (3.0)	20 (2.0)	
Family history of prostate cancer in first-degree relatives			<0.0001
No	549 (75.2)	723 (90.4)	
Yes	181 (24.8)	77 (9.6)	
Educational level			0.27
Less than high school	446 (54.5)	508 (57.9)	
High school graduate	113 (13.8)	110 (12.5)	
College graduate	260 (31.7)	260 (29.6)	
Smoking status			0.21
Never smoker	240 (29.3)	246 (28.0)	
Former smoker	455 (55.7)	476 (54.1)	
Current smoker	123 (15.0)	157 (17.9)	
Alcohol drinking^2^			0.22
Never	72 (8.8)	84 (9.6)	
Low drinkers	565 (69.0)	573 (65.2)	
Heavy drinkers	182 (22.2)	222 (25.3)	
Physical activity			0.03
Less than one hour/week during at least one year	191 (23.4)	177 (20.1)	
Less than 3 hours a week during less than 19 years	108 (13.2)	126 (14.3)	
More than 3 hours a week during less than 19 years	131 (16.0)	140 (15.9)	
Less than 3 hours a week during more than 19 years	180 (22.0)	243 (27.7)	
More than 3 hours a week during more than 19 years	208 (25.4)	193 (22.0)	
Personal history of cardiovascular disease^3^			0.64
No	734 (89.9)	776 (89.0)	
Yes	82 (10.1)	96 (11.0)	
Diabetes history			0.63
No	710 (86.8)	750 (85.7)	
Yes	108 (13.2)	125 (14.3)	
Treated	96 (89.7)	115 (92.0)	

^1^Adjusted for age (excepted for age).

^2^Never: Less than once a month during one year; Low drinkers: at least once a month during one year and zero or one positive answer to the CAGE questionnaire; Heavy drinkers: at least once a month during one year and two or more positive answer to the CAGE questionnaire.

^3^Myocardial infarction, angina pectoris, stroke.

Associations between anthropometric indicators (height, BMI, WC and WHR) and prostate cancer risk, overall and according to PCa aggressiveness, are shown in Table 2. A height between 172 and 177 cm was associated with an increase of prostate cancer risk compared to a height between 168 and 172 cm (OR 1.38, 95% CI 1.04–1.82), without any trend in the risk (*p* = 0.58). We did not found any significant association with BMI, either for overall PCa or for aggressive PCa. We observed a slight, but not significant increased risk of PCa for men with a WC above 94 cm (OR 1.20, 95% CI 0.92–1.56) and for men with a WHR greater or equal to 0.95 (OR 1.30, 95% CI 1.00–1.70 for WHR between 0.95 and 1.00, OR 1.25, 95% CI 0.96–1.61 for WHR above 1.00). Associations between WC and PCa were more pronounced for aggressive PCa (OR 1.72, 95% CI 1.07–2.77 for WC between 94 and 102 cm, OR 1.80, 95% CI 1.13–2.88 for WC greater to 102 cm). Associations between WHR and PCa were also more pronounced for aggressive PCa (OR 1.56, 95% CI 1.01–2.42 for WHR above 1.00), while a modest but not significant increase of aggressive PCa risk was observed for WHR between 0.95 and 0.99 (OR 1.33, 95% CI 0.84–2.10).

**Table 2:** Associations between height, body mass index, waist circumference, waist on hip ratio and prostate cancer risk

	Controls	Cases					
		All		Low and intermediate^1^		Aggressive^2^	
	*n* = 879 (%)	*n* = 819 (%)	OR (95% CI)^3^	*n* = 623 (%)	OR (95% CI)^3^	*n* = 183 (%)	OR (95% CI)^3^
Height (cm), measured at interview							
<168	160 (18.8)	156 (19.2)	1.18 [0.86–1.62]	119 (19.3)	1.32 [0.93–1.87]	33 (18.0)	0.80 [0.47–1.36]
168–171	227 (26.6)	191 (23.5)	1.00 reference	141 (22.9)	1.00 reference	48 (26.2)	1.00 reference
172–176	241 (28.2)	256 (31.5)	1.38 [1.04–1.82]	196 (31.8)	1.49 [1.10–2.02]	57 (31.2)	1.11 [0.71–1.73]
≥177	225 (26.4)	209 (25.8)	1.14 [0.85–1.53]	160 (26.0)	1.22 [0.89–1.68]	45 (24.6)	0.92 [0.57–1.48]
			*P trend = 0.58*		*P trend = 0.64*		*P trend = 0.61*
Body Mass Index (kg/m^2^), self-reported (2 years prior diagnosis)							
<25	316 (36.6)	297 (36.7)	1.00 reference	229 (37.3)	1.00 reference	60 (33.3)	1.00 reference
25–29	395 (45.8)	377 (46.7)	0.98 [0.78–1.23]	288 (46.8)	0.95 [0.74–1.21]	85 (47.2)	1.17 [0.80–1.73]
≥30	152 (17.6)	134 (16.6)	0.91 [0.67–1.23]	98 (15.9)	0.86 [0.62–1.20]	35 (19.5)	1.19 [0.73–1.96]
			*P trend = 0.56*		*P trend = 0.38*		*P trend = 0.43*
Body Mass Index (kg/m^2^), measured at interview							
<25	248 (29.1)	231 (28.5)	1.00 reference	172 (27.9)	1.00 reference	53 (29.0)	1.00 reference
25–29	397 (46.6)	399 (49.1)	1.07 [0.84–1.37]	312 (50.7)	1.11 [0.85–1.45]	82 (44.8)	1.02 [0.68–1.54]
≥30	207 (24.3)	182 (22.4)	0.88 [0.66–1.18]	132 (21.4)	0.88 [0.64–1.21]	48 (26.2)	0.97 [0.60–1.56]
			*P trend = 0.45*		*P trend = 0.48*		*P trend = 0.91*
Waist circumference (cm), measured at interview							
≤94	254 (29.7)	209 (25.9)	1.00 reference	168 (27.4)	1.00 reference	35 (19.3)	1.00 reference
95–102	284 (33.1)	290 (35.9)	1.20 [0.92–1.56]	220 (35.8)	1.11 [0.83–1.47]	65 (35.9)	1.72 [1.07–2.77]
>102	319 (37.2)	309 (38.2)	1.20 [0.92–1.56]	226 (36.8)	1.10 [0.83–1.46]	81 (44.8)	1.80 [1.13–2.88]
			*P trend = 0.20*		*P trend = 0.51*		*P trend = 0.02*
Waist-Hip Ratio (WHR)							
<0.95	265 (31.0)	214 (26.5)	1.00 reference	166 (27.1)	1.00 reference	43 (23.8)	1.00 reference
0.95–0.99	272 (31.8)	281 (34.9)	1.30 [1.00–1.70]	218 (35.6)	1.30 [0.98–1.73]	58 (32.0)	1.33 [0.84–2.10]
≥1.00	318 (37.2)	311 (38.6)	1.25 [0.96–1.61]	229 (37.3)	1.18 [0.89–1.56]	80 (44.2)	1.56 [1.01–2.42]
			*P trend = 0.12*		*P trend = 0.29*		*P trend = 0.04*

^1^Gleason ≤ 7 (3+4)

^2^Gleason ≥ 7 (4+3)

^3^ORs adjusted for age, family history of cancer at first degree, ethnicity.

Previous analyses were also adjusted for BMI in addition to age, family history of cancer and ethnicity (Table 3). Associations were more pronounced after adjustment for BMI (OR 1.43, 95% CI 1.07–1.91 for WC between 94 and 102 cm, OR 1.38, 95% CI 1.05–1.81 for WHR between 0.95 and 1.00). Associations regarding abdominal obesity indicators, adjusted for BMI, were also more pronounced in men with aggressive prostate cancer, either for WC > 94 cm (OR 2.20, 95% CI 1.32–3.69 for WC between 94 and 102 cm, OR 3.27, 95% CI 1.70–6.30 for WC greater to 102 cm) or WHR ≥ 0.95 (OR 1.40, 95% CI 0.87–2.23 for WHR between 0.95 and 1.00, OR 1.77, 95% CI 1.09–2.87 for WHR above 1.00).

**Table 3:** Associations between height, waist circumference, waist on hip ratio and prostate cancer risk, adjusted for body mass index

	Controls	Cases					
	*n* = 879 (%)	All		Low and intermediate^1^		Aggressive^2^	
		*n* = 819 (%)	OR (95% CI)^3^	*n* = 623 (%)	OR (95% CI)^3^	*n* = 183 (%)	OR (95% CI)^3^
Height (cm), measured at interview							
<168	160 (18.8)	156 (19.2)	1.19 [0.86–1.63]	119 (19.3)	1.33 [0.94–1.88]	33 (18.0)	0.80 [0.47–1.36]
168–171	227 (26.6)	191 (23.5)	1.00 reference	141 (22.9)	1.00 reference	48 (26.2)	1.00 reference
172–176	241 (28.2)	256 (31.5)	1.38 [1.04–1.82]	196 (31.8)	1.49 [1.10–2.02]	57 (31.2)	1.11 [0.71–1.73]
≥177	225 (26.4)	209 (25.8)	1.14 [0.85–1.53]	160 (26.0)	1.22 [0.89–1.68]	45 (24.6)	0.92 [0.57–1.48]
			*P trend = 0.58*		*P trend = 0.65*		*P trend = 0.61*
Waist circumference (cm), measured at interview							
≤94	254 (29.7)	209 (25.9)	1.00 reference	168 (27.4)	1.00 reference	35 (19.3)	1.00 reference
95–102	284 (33.1)	290 (35.9)	1.43 [1.07–1.91]	220 (35.8)	1.30 [0.95–1.77]	65 (35.9)	2.20 [1.32–3.69]
>102	319 (37.2)	309 (38.2)	1.86 [1.26–2.72]	226 (36.8)	1.63 [1.08–2.46]	81 (44.8)	3.27 [1.70–6.30]
			*P trend = 0.002*		*P trend = 0.02*		*P trend = 0.0004*
Waist-Hip Ratio (WHR)							
<0.95	265 (31.0)	214 (26.5)	1.00 reference	166 (27.1)	1.00 reference	43 (23.8)	1.00 reference
0.95–0.99	272 (31.8)	281 (37.9)	1.38 [1.05–1.81]	218 (35.6)	1.38 [1.03–1.84]	58 (32.0)	1.40 [0.87–2.23]
≥1.00	318 (37.2)	311 (38.6)	1.43 [1.07–1.90]	229 (37.3)	1.35 [0.99–1.84]	80 (44.2)	1.77 [1.09–2.87]
			*P trend = 0.02*		*P trend = 0.07*		*P trend = 0.02*

^1^Gleason ≤ 7 (3+4)

^2^Gleason ≥ 7 (4+3)

^3^ORs adjusted for age, family history of cancer at first degree, ethnicity, body mass index.

Associations between anthropometric indicators and prostate cancer risk, stratified on BMI (cut-point of 25 kg/m^2^) are presented in Table 4. A higher risk of overall PCa was observed for men with a BMI under 25 kg/m^2^ compared to men having a BMI over 25 kg/m^2^, either for WC > 94 cm (OR 1.60, 95% CI 1.03–2.48 vs OR 1.13, 95% CI 0.76–1.67) or WHR ≥ 0.95 (OR 1.75, 95% CI 1.17–2.60 vs OR 1.10, 95% CI 0.81–1.49). A higher risk of low/intermediate PCa was also observed for normal weight men compared to overweight/obese men, either for WC > 94 cm (OR 1.49, 95% CI 0.92–2.40 vs OR 0.94, 95% CI 0.62–1.41) or WHR ≥ 0.95 (OR 1.78, 95% CI 1.15–2.75 vs OR 1.02, 95% CI 0.74–1.42). Associations were more pronounced for aggressive PCa in comparison to overall PCa (OR 2.03, 95% CI 1.02–4.03 vs 1.60, 95% CI 1.03–2.48 for men with BMI < 25 kg/m^2^ and WC > 94; OR 3.50, 95% CI 1.25–9.83 vs 1.13, 95% CI 0.76–1.67 for men with BMI ≥ 25 kg/m^2^ and WC > 94). Nevertheless, interactions were not significant for WC (*p* = 0.23) and close to significance for WHR (*p* = 0.07).

**Table 4:** Associations between waist circumference, waist on hip ratio, and prostate cancer risk, stratified on body mass index

	Controls	Cases					
		All		Low and intermediate^1^		Aggressive^2^	
	*n* = 879 (%)	*n* = 819 (%)	OR (95% CI)^3^	*n* = 623 (%)	OR (95% CI)^3^	*n* = 183 (%)	OR (95% CI)^3^
**BMI < 25 kg/m**^2^							
Waist circumference (cm)							
≤94	183 (73.8)	151 (65.7)	1.00 reference	117 (68.0)	1.00 reference	30 (57.7)	1.00 reference
>94	65 (26.2)	79 (34.3)	1.60 [1.03–2.48]	55 (32.0)	1.49 [0.92–2.40]	22 (42.3)	2.03 [1.02–4.03]
95–102	58 (23.4)	71 (30.9)	1.65 [1.05–2.58]	49 (28.5)	1.53 [0.94–2.50]	20 (38.5)	2.09 [1.04–4.20]
>102	7 (2.8)	8 (3.5)	1.07 [0.27–4.18]	6 (3.5)	1.04 [0.23–4.60]	2 (3.8)	1.35 [0.15–12.42]
Waist-Hip Ratio (WHR)							
<0.95	143 (57.7)	108 (47.0)	1.00 reference	81 (47.1)	1.00 reference	25 (48.1)	1.00 reference
≥0.95	105 (42.3)	122 (53.0)	1.75 [1.17–2.60]	91 (52.9)	1.78 [1.15–2.75]	27 (51.9)	1.54 [0.80–2.99]
0.95–0.99	63 (25.4)	81 (35.2)	1.92 [1.23–3.02]	59 (34.3)	1.94 [1.19–3.17]	19 (36.5)	1.75 [0.84–3.64]
≥ 1.00	42 (16.9)	41 (17.8)	1.45 [0.83–2.54]	32 (18.6)	1.51 [0.82–2.77]	8 (15.4)	1.22 [0.47–3.13]
**BMI ≥ 25 kg/m**^**2**^							
Waist circumference (cm)							
≤94	71 (11.8)	58 (10.0)	1.00 reference	51 (11.5)	1.00 reference	5 (3.9)	1.00 reference
>94	533 (88.2)	520 (90.0)	1.13 [0.76–1.67]	391 (88.5)	0.94 [0.62–1.41]	124 (96.1)	3.50 [1.25–9.83]
95–102	225 (37.3)	219 (37.9)	1.07 [0.70–1.62]	171 (38.7)	0.89 [0.58–1.39]	45 (34.9)	3.15 [1.09–9.12]
>102	308 (51.0)	301 (52.1)	1.18 [0.78–1.77]	220 (49.8)	0.97 [0.63–1.48]	79 (61.2)	3.77 [1.32–10.72]
Waist-Hip Ratio (WHR)							
<0.95	122 (20.2)	106 (18.4)	1.00 reference	85 (19.3)	1.00 reference	18 (14.0)	1.00 reference
≥0.95	482 (79.8)	470 (81.6)	1.10 [0.81–1.49]	356 (80.7)	1.02 [0.74–1.42]	111 (86.0)	1.52 [0.86–2.69]
0.95–0.99	209 (34.6)	200 (34.7)	1.06 [0.75–1.49]	159 (36.0)	1.03 [0.71–1.48]	39 (30.2)	1.26 [0.67–2.38]
≥1.00	273 (45.2)	270 (46.9)	1.13 [0.81–1.57]	197 (44.7)	1.02 [0.72–1.45]	72 (55.8)	1.73 [0.96–3.14]

^1^Gleason ≤ 7 (3+4)

^2^Gleason ≥ 7 (4+3)

^3^ORs adjusted for age, family history of cancer at first degree, ethnicity.

Sensitivity analyses limited to Caucasians revealed similar results (data not shown).

## DISCUSSION

Our study showed a positive association between anthropometric indicators assessing abdominal obesity and risk of PCa in a large population-based study. While a modest but not significant association was observed for WC or WHR and PCa before adjustment for BMI, excess risk of PCa was observed after adjustment for BMI, with a dose-response trend between these anthropometric indicators and PCa risk. Moreover, we observed that associations were more pronounced for aggressive PCa. However, we did not observe any association with BMI or height and PCa.

Our results are in line with few studies that considered abdominal obesity indicators (WC and WHR) exclusively [32] and both abdominal and global obesity indicators (BMI) [10, 33–35]. Studies that have examined abdominal obesity indicators taking simultaneously into account BMI also observed a significant increase of PCa risk overall with abdominal obesity (WC and WHR indicators) among individuals with a BMI under 25 kg/m^2^ [10, 16, 36]. Based on the literature, we hypothesized that WC adjusted for BMI may be a better predictor of intra-abdominal fat mass than WC alone [37, 38]. Although accurate quantification of body fat compartments requires imaging techniques such as magnetic resonance imaging (MRI) and computed tomography (CT) [39, 40], these techniques are not feasible in large-scale studies due to their expensive cost and complexity. However, WC and WHR are good surrogate markers to assess regional adiposity as they well correlate with laboratory-based measures of adiposity using MRI or CT [41–47].

Taking into account several anthropometric indicators conjointly may also better predict prostate cancer risk [10]. Associations with abdominal obesity indicators were more pronounced in aggressive PCa, which is in accordance with the literature [10–12, 16, 18, 32, 33, 48].

Several mechanisms may underlie the association between obesity and PCa through metabolic, hormonal and inflammatory pathways. In particular, it is known that obese men tend to have lower levels of androgens and adiponectin as well as higher levels of insulin and insulin-like growth factor (IGF-I) [21, 22]. Evidence suggest that high insulin and high circulating IGF-I levels are associated with an increased risk of PCa [23–25]. Obesity is also associated with a lower concentration of free testosterone [49, 50], resulting in the growth of aggressive prostate tumors [51]. In addition, obesity is associated with a low-grade chronic inflammatory state and inflammation may be involved in PCa occurrence [29, 52].

Our results are based on a large population-based case-control study specifically designed to assess environmental and genetic factors in prostate cancer occurrence. Cases were identified in all cancer hospitals, either public or private, that recruited prostate cancer patients in the département of Hérault. In 2011, the Hérault Cancer Registry observed 770 new cases of prostate cancer in men aged less than 75 years old. Considering that the number of cases observed in 2011 was similar during the study period, approximately 1150 new cases were expected during 2012–2013 [53]. We identified 1098 eligible cases over the study period suggesting that the recruitment of cases in the EPICAP study was quite exhaustive, thus limiting a potential selection bias. Controls were randomly selected from the general population of the département of Hérault using quotas defined for age (5 years) and SES. The age distribution of the controls reflects the age distribution of the cases. In order to avoid selection bias, the SES distribution of the control group reflects the SES distribution of the entire département of Hérault to yield the control group similar to the general population of men of the same age in terms of SES. After the selection process, the distribution by SES between our control group and the male general population of the département of Hérault has been compared and no significant difference has been found, suggesting that no major selection bias by SES had occurred.

To minimize differential classification bias that can persist in case-control studies, data were collected by the same clinical research nurses for cases and controls, and under the same conditions using a standardized questionnaire.

To minimize possible differential misclassification bias, we rather used anthropometric indicators that have been measured at interview by the nurses than the self-declared indicators. Indeed, self-declared BMI and measured BMI were different in extreme categories (< 25 kg/m^2^ and ≥ 30 kg/m^2^).

Our results remained unchanged after adjustment for potential major confounding factors such as educational level, physical activity, and smoking status, thus limiting potential confounding.

In conclusion, our results support a role of abdominal obesity in PCa risk, and particularly aggressive PCa, while BMI itself would not. Furthermore, our results also suggest that WC appears to be a better measure of abdominal obesity than WHR. The association between obesity and aggressive PCa is notably pertinent due to the large numbers of men affected by both diseases. The identification of abdominal obesity as a risk factor for aggressive PCa would be very important from a public health point of view and may provide new prevention strategies.

## MATERIALS AND METHODS

### Study population

EPICAP is a population-based case-control study carried out in the département of Hérault, a well delimited geographic area in the South of France. Details of the EPICAP objectives and study design have been previously described [54]. In brief, cases were men newly diagnosed for PCa in 2012–2013, aged under 75 and living in the département of Hérault at time of diagnosis. Controls were men randomly selected from the general population, frequency-matched to the cases by 5-year age group, living in the same département as the cases and with no history of PCa at the time of inclusion. Quotas by socioeconomic status (SES) were set a priori to control for potential selection bias arising from differential participation rates across SES categories. These quotas were computed from the census data available in the département of Hérault, in order to obtain a distribution by SES among controls identical to the SES distribution among general male population, conditionally to age.

Overall, 819 incident prostate cancer cases and 879 population-based controls were recruited with a participation rate of 75% and 79%, respectively. All participants included in the study provided a written consent. The EPICAP study was approved by the review board of the French national institute of health and medical research (INSERM, n°01–040, November 2010) and authorized by the French data protection authority (CNIL n°910485, April 2011).

### Data collection

Cases and controls provided information about socio-demographic characteristics, occupational and residential history, lifestyle and leisure activities, personal and family medical history and anthropometric factors using a face-to-face standardized computerized questionnaire (CAPI, Computer Assisted Personal Interview) realized by research clinical nurses.

Anthropometric factors of interest included height, weight, waist and hip circumferences. Self-reported height at 20 years old and weight two years before the reference date (i.e., date of diagnosis for cases or date of interview for controls) were asked in the questionnaire and height, weight, waist and hip circumferences were measured at interview by the research clinical nurses.

For cases, medical data such as Gleason scores, Prostate Specific Antigen (PSA) levels, and tumor stage at diagnosis were extracted from patient’s medical records and validated by the Hérault Cancer Registry.

### Statistical analysis

For alcohol consumption, men were asked during interview whether they drink more than once a month during one year (No/Yes). For those who answered “Yes”, the level of alcohol consumption was assessed using the CAGE questionnaire [55]. Alcohol consumption has been categorized into three classes: never drinkers (less than once a month during one year), low drinkers (at least once a month during one year and zero or one positive answer to the CAGE questionnaire), and heavy drinkers (at least once a month during one year and two or more positive answers to the CAGE questionnaire).

Physical activity level was categorized using the median number of years of sport practice and the median number of hours per week of practice for the same sport, calculated in the control population. If the participant reported practicing more than one sport during his entire life, we used the sport he practiced the longest. Therefore, physical activity level was categorized into five categories classes: no activity (less than one hour per week during at least one year), less than 3 hours per week during less than 19 years, more than 3 hours per week during less than 19 years, less than 3 hours per week during more than 19 years, and more than 3 hours per week during more than 19 years.

Height was categorized at the quartile values of the control series. BMI was calculated as either self-reported or measured weight divided by the square of the height. We categorized BMI according to the definition of the World Health Organization (WHO) into three classes: under-weight and normal weight (BMI < 25 kg/m^2^), overweight (BMI: 25–29.9 kg/m^2^), and obese (BMI ≥ 30 kg/m^2^). All analyses used the BMI computed from the measured weight and height.

WC was measured horizontally around the waist at the level of the navel and hip measurement was taken at the widest lateral extension of the hips. WHR was calculated by dividing the WC by the hip circumference. We used the WHO recommended cut-points related to an increase risk of metabolic and cardiovascular diseases for European populations [56]. For WC, we used 94 and 102 centimeters cut-points; for WHR, we used 0.95 and 1.00 cut-points.

Unconditional logistic regression models were used to estimate odds ratios (ORs) and their 95% Confidence Interval (CI). Analyses were systematically adjusted for age (5-year groups), family history of prostate cancer in first-degree relatives and ethnic origin (caucasians, others). Analyses were adjusted for other potential confounding factors such as educational level or physical activity.

Analyses with WC and WHR were also adjusted for BMI (continuously) to better capture abdominal obesity.

Separate analyses were also conducted by prostate cancer aggressiveness according to the Gleason score at diagnosis (low or intermediate aggressiveness: Gleason score < 7 or Gleason score = 7 including subjects for whom the two most commonly represented grades in the tumor are 3 + 4, high aggressiveness: Gleason score ≥ 8 or Gleason score = 7 including subjects for whom the two grades are 4 + 3)).

Seeking for interaction with BMI, we performed stratified analyses splitting EPICAP population according to BMI overweight cut-point (< 25 kg/m^2^ / ≥ 25 kg/m^2^). *P*-values testing for interaction were based on the Wald test.

All statistical analyses were performed using SAS (version 9.4; SAS Institute Inc., Cary, NC, USA).

## Abbreviations

BMI: Body Mass index
CI: Confidence Interval
cm: centimeters
IGF: Insulin-like Growth Factor
OR: Odds Ratio
PCa: Prostate Cancer
PSA: Prostate Specific Antigen
RR: Relative Risk
SES: Socioeconomic Status
WC: Waist Circumference
WHO: World Health Organization
WHR: Waist-Hip Ratio

## References

[1] Torre LA, Bray F, Siegel RL, Ferlay J, Lortet-Tieulent J, Jemal A (2015). Global cancer statistics, 2012. CA Cancer J Clin.

[2] Grosclaude P, Belot A, Daubisse Marliac L, Remontet L, Leone N, Bossard N, Velten M, Francim réseau (2015). Prostate cancer incidence and mortality tr ends in France from 1980 to 2011. [Article in French] Progres En Urol J Assoc Francaise Urol Soc Francaise Urol.

[3] Leone N, Voirin N, Roche L, Binder-Foucard F, Woronoff AS, Delafosse P, Remontet L, Bossard N, Uhry Z (2015). Projection de l’incidence et de la mortalité par cancer en France métropolitaine en 2015. http://invs.santepubliquefrance.fr/content/download/119139/419229/version/1/file/Cancer_Projection2015_PROSTATE.pdf.

[4] Boeing H (2013). Obesity and cancer--the update 2013. Best Pract Res Clin Endocrinol Metab.

[5] Renehan AG, Tyson M, Egger M, Heller RF, Zwahlen M (2008). Body-mass index and incidence of cancer: a systematic review and meta-analysis of prospective observational studies. Lancet.

[6] Bergström A, Pisani P, Tenet V, Wolk A, Adami HO (2001). Overweight as an avoidable cause of cancer in Europe. Int J Cancer.

[7] Discacciati A, Orsini N, Wolk A (2012). Body mass index and incidence of localized and advanced prostate cancer--a dose-response meta-analysis of prospective studies. Ann Oncol.

[8] MacInnis RJ, English DR (2006). Body size and composition and prostate cancer risk: systematic review and meta-regression analysis. Cancer Causes Control.

[9] Harding JL, Shaw JE, Anstey KJ, Adams R, Balkau B, Brennan-Olsen SL, Briffa T, Davis TME, Davis WA, Dobson A, Flicker L, Giles G, Grant J (2015). Comparison of anthropometric measures as predictors of cancer incidence: A pooled collaborative analysis of 11 Australian cohorts. Int J Cancer.

[10] Boehm K, Sun M, Larcher A, Blanc-Lapierre A, Schiffmann J, Graefen M, Sosa J, Saad F, Parent MÉ, Karakiewicz PI (2015). Waist circumference, waist-hip ratio, body mass index, and prostate cancer risk: results from the North-American case-control study Prostate Cancer & Environment Study. Urol Oncol.

[11] Guerrios-Rivera L, Howard L, Frank J, De Hoedt A, Beverly D, Grant DJ, Hoyo C, Freedland SJ (2017). Is Body Mass Index the Best Adiposity Measure for Prostate Cancer Risk? Results From a Veterans Affairs Biopsy Cohort. Urology.

[12] Tang B, Han CT, Zhang GM, Zhang CZ, Yang WY, Shen Y, Vidal AC, Freedland SJ, Zhu Y, Ye DW (2017). Waist-hip Ratio (WHR), a Better Predictor for Prostate Cancer than Body Mass Index (BMI): Results from a Chinese Hospital-based Biopsy Cohort. Sci Rep.

[13] Dimitropoulou P, Martin RM, Turner EL, Lane JA, Gilbert R, Davis M, Donovan JL, Hamdy FC, Neal DE (2011). Association of obesity with prostate cancer: a case-control study within the population-based PSA testing phase of the ProtecT study. Br J Cancer.

[14] Fowke JH, Motley S, Dai Q, Concepcion R, Barocas DA (2013). Association between biomarkers of obesity and risk of high-grade prostatic intraepithelial neoplasia and prostate cancer—evidence of effect modification by prostate size. Cancer Lett.

[15] Hsing AW, Deng J, Sesterhenn IA, Mostofi FK, Stanczyk FZ, Benichou J, Xie T, Gao YT (2000). Body size and prostate cancer: a population-based case-control study in China. Cancer Epidemiol Biomarkers Prev.

[16] De Nunzio C, Albisinni S, Freedland SJ, Miano L, Cindolo L, Finazzi Agrò E, Autorino R, De Sio M, Schips L, Tubaro A (2013). Abdominal obesity as risk factor for prostate cancer diagnosis and high grade disease: a prospective multicenter Italian cohort study. Urol Oncol.

[17] Gong Z, Neuhouser ML, Goodman PJ, Albanes D, Chi C, Hsing AW, Lippman SM, Platz EA, Pollak MN, Thompson IM, Kristal AR (2006). Obesity, diabetes, and risk of prostate cancer: results from the prostate cancer prevention trial. Cancer Epidemiol Biomarkers Prev.

[18] Perez-Cornago A, Appleby PN, Pischon T, Tsilidis KK, Tjønneland A, Olsen A, Overvad K, Kaaks R, Kühn T, Boeing H, Steffen A, Trichopoulou A, Lagiou P (2017). Tall height and obesity are associated with an increased risk of aggressive prostate cancer: results from the EPIC cohort study. BMC Med.

[19] Su LJ, Arab L, Steck SE, Fontham ETH, Schroeder JC, Bensen JT, Mohler JL (2011). Obesity and prostate cancer aggressiveness among African and Caucasian Americans in a population-based study. Cancer Epidemiol Biomarkers Prev.

[20] Kruk J, Czerniak U (2013). Physical activity and its relation to cancer risk: updating the evidence. Asian Pac J Cancer Prev.

[21] Giovannucci E, Michaud D (2007). The role of obesity and related metabolic disturbances in cancers of the colon, prostate, and pancreas. Gastroenterology.

[22] Continuous Update Project Report: Diet, Nutrition, Physical Activity, and Prostate Cancer (2014). World Cancer Research Fund. http://www.wcrf.org/sites/default/files/Prostate-Cancer-2014-Report.pdf.

[23] Cao Y, Nimptsch K, Shui IM, Platz EA, Wu K, Pollak MN, Kenfield SA, Stampfer MJ, Giovannucci EL (2015). Prediagnostic plasma IGFBP-1, IGF-1 and risk of prostate cancer. Int J Cancer.

[24] Roddam AW, Allen NE, Appleby P, Key TJ, Ferrucci L, Carter HB, Metter EJ, Chen C, Weiss NS, Fitzpatrick A, Hsing AW, Lacey JV, Helzlsouer K (2008). Insulin-like growth factors, their binding proteins, and prostate cancer risk: analysis of individual patient data from 12 prospective studies. Ann Intern Med.

[25] Nandeesha H (2009). Insulin: a novel agent in the pathogenesis of prostate cancer. Int Urol Nephrol.

[26] Balkwill F, Mantovani A (2001). Inflammation and cancer: back to Virchow?. Lancet.

[27] Coussens LM, Werb Z (2002). Inflammation and cancer. Nature.

[28] De Marzo AM, Marchi VL, Epstein JI, Nelson WG (1999). Proliferative inflammatory atrophy of the prostate: implications for prostatic carcinogenesis. Am J Pathol.

[29] De Marzo AM, Platz EA, Sutcliffe S, Xu J, Grönberg H, Drake CG, Nakai Y, Isaacs WB, Nelson WG (2007). Inflammation in prostate carcinogenesis. Nat Rev Cancer.

[30] Mantovani A, Allavena P, Sica A, Balkwill F (2008). Cancer-related inflammation. Nature.

[31] Thapa D, Ghosh R (2015). Chronic inflammatory mediators enhance prostate cancer development and progression. Biochem Pharmacol.

[32] Nemesure B, Wu SY, Hennis A, Leske MC, Prostate Cancer in a Black Population (PCBP) Study Group (2012). Central adiposity and Prostate Cancer in a Black Population. Cancer Epidemiol Biomarkers Prev.

[33] Jackson MD, Walker SP, Simpson CM, McFarlane-Anderson N, Bennett FI, Coard KC, Aiken WD, Tulloch T, Paul TJ, Wan RL (2010). Body size and risk of prostate cancer in Jamaican men. Cancer Causes Control.

[34] Esposito K, Chiodini P, Capuano A, Bellastella G, Maiorino MI, Parretta E, Lenzi A, Giugliano D (2013). Effect of metabolic syndrome and its components on prostate cancer risk: meta-analysis. J Endocrinol Invest.

[35] Wallström P, Bjartell A, Gullberg B, Olsson H, Wirfält E (2009). A prospective Swedish study on body size, body composition, diabetes, and prostate cancer risk. Br J Cancer.

[36] Pischon T, Boeing H, Weikert S, Allen N, Key T, Johnsen NF, Tjønneland A, Severinsen MT, Overvad K, Rohrmann S, Kaaks R, Trichopoulou A, Zoi G (2008). Body size and risk of prostate cancer in the European prospective investigation into cancer and nutrition. Cancer Epidemiol Biomarkers Prev.

[37] Berentzen TL, Ängquist L, Kotronen A, Borra R, Yki-Järvinen H, Iozzo P, Parkkola R, Nuutila P, Ross R, Allison DB, Heymsfield SB, Overvad K, Sørensen TI, Jakobsen MU (2012). Waist circumference adjusted for body mass index and intra-abdominal fat mass. PLoS One.

[38] Arner P (1998). Not all fat is alike. Lancet Lond Engl.

[39] Abate N, Burns D, Peshock RM, Garg A, Grundy SM (1994). Estimation of adipose tissue mass by magnetic resonance imaging: validation against dissection in human cadavers. J Lipid Res.

[40] Deurenberg P, Yap M (1999). The assessment of obesity: methods for measuring body fat and global prevalence of obesity. Best Pract Res Clin Endocrinol Metab.

[41] Chan DC, Watts GF, Barrett PHR, Burke V (2003). Waist circumference, waist-to-hip ratio and body mass index as predictors of adipose tissue compartments in men. QJM Mon J Assoc Physicians.

[42] Kamel EG, McNeill G, Van Wijk MC (2000). Usefulness of anthropometry and DXA in predicting intra-abdominal fat in obese men and women. Obes Res.

[43] Owens S, Litaker M, Allison J, Riggs S, Ferguson M, Gutin B (1999). Prediction of visceral adipose tissue from simple anthropometric measurements in youths with obesity. Obes Res.

[44] Ferland M, Després JP, Tremblay A, Pinault S, Nadeau A, Moorjani S, Lupien PJ, Thériault G, Bouchard C (1989). Assessment of adipose tissue distribution by computed axial tomography in obese women: association with body density and anthropometric measurements. Br J Nutr.

[45] Ross R, Shaw KD, Martel Y, de Guise J, Avruch L (1993). Adipose tissue distribution measured by magnetic resonance imaging in obese women. Am J Clin Nutr.

[46] Janssen I, Heymsfield SB, Allison DB, Kotler DP, Ross R (2002). Body mass index and waist circumference independently contribute to the prediction of nonabdominal, abdominal subcutaneous, and visceral fat. Am J Clin Nutr.

[47] Rankinen T, Kim SY, Pérusse L, Després JP, Bouchard C (1999). The prediction of abdominal visceral fat level from body composition and anthropometry: ROC analysis. Int J Obes Relat Metab Disord.

[48] Fowke JH, Motley SS, Concepcion RS, Penson DF, Barocas DA (2012). Obesity, body composition, and prostate cancer. BMC Cancer.

[49] Allott EH, Masko EM, Freedland SJ (2013). Obesity and prostate cancer: weighing the evidence. Eur Urol.

[50] Kelly DM, Jones TH (2015). Testosterone and obesity. Obes Rev Off J Int Assoc Study Obes.

[51] Platz EA, Leitzmann MF, Rifai N, Kantoff PW, Chen YC, Stampfer MJ, Willett WC, Giovannucci E (2005). Sex steroid hormones and the androgen receptor gene CAG repeat and subsequent risk of prostate cancer in the prostate-sp ecific antigen era. Cancer Epidemiol Biomarkers Prev.

[52] Hursting SD, Hursting MJ (2012). Growth signals, inflammation, and vascular perturbations: mechanistic links between obesity, metabolic syndrome, and cancer. Arterioscler Thromb Vasc Biol.

[53] Registre des tumeurs de l’herault. 2018. Available from: http://www.registre-tumeurs-herault.fr/

[54] Menegaux F, Anger A, Randrianasolo H, Mulot C, Laurent-Puig P, Iborra F, Bringer JP, Leizour B, Thuret R, Lamy PJ, Rébillard X, Trétarre B, EPICAP Study Group (2014). Epidemiological study of prostate cancer (EPICAP): a population-based case-control study in France. BMC Cancer.

[55] Ewing JA (1984). Detecting alcoholism. The CAGE questionnaire. JAMA.

[56] Hu MB, Liu SH, Jiang HW, Bai PD, Ding Q (2014). Obesity affects the biopsy-mediated detection of prostate cancer, particularly high-grade prostate cancer: a dose-response meta-analysis of 29,464 patients. PloS One.

